# [Corrigendum] MCMV triggers ROS/NLRP3-associated inflammasome activation in the inner ear of mice and cultured spiral ganglion neurons, contributing to sensorineural hearing loss

**DOI:** 10.3892/ijmm.2025.5514

**Published:** 2025-03-05

**Authors:** Wei Zhuang, Caiji Wang, Xi Shi, Shiwei Qiu, Shili Zhang, Bing Xu, Min Chen, Wen Jiang, Hongyan Dong, Yuehua Qiao

Int J Mol Med 41: 3448-3456, 2018; DOI: 10.3892/ijmm.2018.3539

Subsequently to the publication of this article, the authors have contacted the Editorial Office to inform us that Fig. 6 on p. 3454 was inadvertently assembled incorrectly; essentially, the wrong immunofluorescence data were featured for the 'Control' experiment in Fig. 6A. The revised version of Fig. 6, now showing the correct data for the Control experiment in Fig. 6A, is shown on the next page. Note that this error did not affect either the results or the conclusions reported in this paper. The authors are grateful to the Editor of *International Journal of Molecular Medicine* for allowing them the opportunity to publish this Corrigendum, and apologize both to the Editor and to readership for any inconvenience caused.

## Figures and Tables

**Figure 6:**
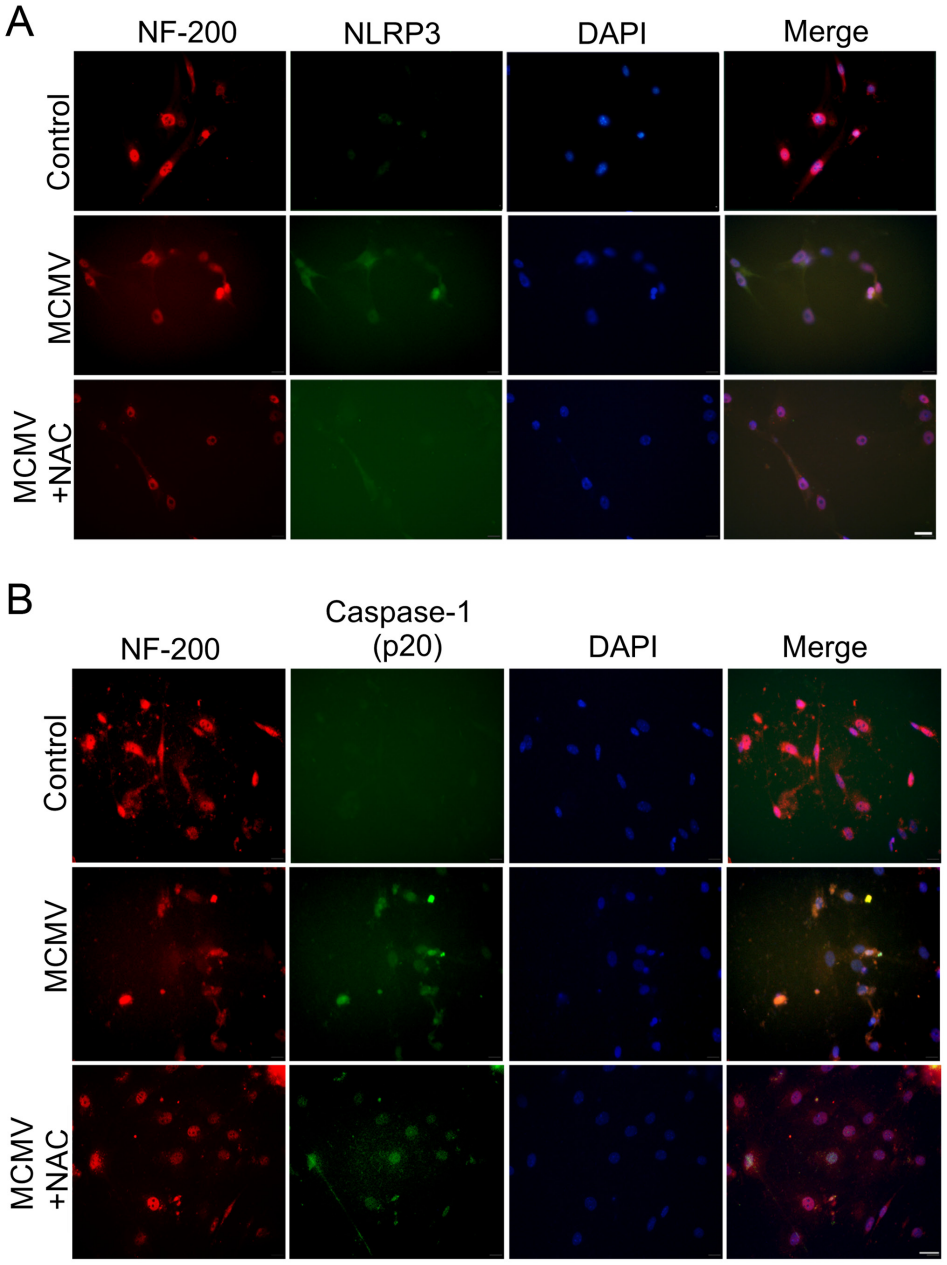
MCMV induces NLRP3 inflammasome activation in cultured SGN. (A) Immunofluorescence analysis of NLRP3 in cultured SGN; immunohistochemical staining for NF-200 (red) as a marker for SGN and NLRP3 (green). (B) Immunofluorescence analysis of caspase-1 (p20) in cultured SGN; immunohistochemical staining for NF-200 (red) and caspase-1 (green). Nuclear counterstain was performed with DAPI (blue; scale bar, 50 µm). SGN, spiral ganglion neurons; NLRP3, nucleotide-binding oligomerization domain-like receptor protein 3; MCMV, murine congenital cytomegalovirus; NeuN, neuronal nuclei; NF, neurofilament; NAC, N-acetyl-L-cysteine.

